# Co-Gelation of Pumpkin-Seed Protein with Egg-White Protein

**DOI:** 10.3390/foods12102030

**Published:** 2023-05-17

**Authors:** Marta Tomczyńska-Mleko, Konrad Terpiłowski, Salvador Pérez-Huertas, Viktoria Sapiga, Galina Polischuk, Bartosz Sołowiej, Maciej Nastaj, Marta Wesołowska-Trojanowska, Stanisław Mleko

**Affiliations:** 1 Institute of Plant Genetics, Breeding and Biotechnology, University of Life Sciences in Lublin, Akademicka 15 Str., 20-950 Lublin, Poland; martamleko@tlen.pl; 2 Department of Interfacial Phenomena, Maria Curie Skłodowska University, Maria Curie-Skłodowska 3 Sq., 20-031 Lublin, Poland; 3 Department of Chemical Engineering, University of Granada, S/N, 18071 Granada, Spain; shuertas@ujaen.es; 4 Department of Technology Milk and Dairy Products, National University of Food Technologies, 68 Volodymyrska Str., 01601 Kyiv, Ukraine; vika.sapiga1904@ukr.net (V.S.); milknuft@i.ua (G.P.); 5 Department of Dairy Technology and Functional Foods, University of Life Sciences in Lublin, Skromna 8, 20-704 Lublin, Poland; bartosz.solowiej@up.lublin.pl (B.S.); maciej.nastaj@up.lublin.pl (M.N.); dairywhey@tlen.pl (S.M.); 6 Department of Biotechnology, Microbiology and Human Nutrition, University of Life Sciences in Lublin, Skromna 8, 20-704 Lublin, Poland; marta.wesolowska-trojanowska@up.lublin.pl

**Keywords:** egg white, pumpkin, protein, gelation, rheology, texture, microstructure, FTIR

## Abstract

The aim of this study was to investigate the gelation process of binary mixes of pumpkin-seed and egg-white proteins. The substitution of pumpkin-seed proteins with egg-white proteins improved the rheological properties of the obtained gels, i.e., a higher storage modulus, lower tangent delta, and larger ultrasound viscosity and hardness. Gels with a larger egg-white protein content were more elastic and more resistant to breaking structure. A higher concentration of pumpkin-seed protein changed the gel microstructure to a rougher and more particulate one. The microstructure was less homogenous, with a tendency to break at the pumpkin/egg-white protein gel interface. The decrease in the intensity of the amide II band with an increase in the pumpkin-seed protein concentration showed that the secondary structure of this protein evolved more toward a linear amino acid chain compared with the egg-white protein, which could have an impact on the microstructure. The supplementation of pumpkin-seed proteins with egg-white proteins caused a decrease in water activity from 0.985 to 0.928, which had important implications for the microbiological stability of the obtained gels. Strong correlations were found between the water activity and rheological properties of the gels; an improvement of their rheological properties resulted in a decrease in water activity. The supplementation of pumpkin-seed proteins with egg-white proteins resulted in more homogenous gels with a stronger microstructure and better water binding.

## 1. Introduction

Protein-based gels are widely used in the food industry due to their ability to impart desirable textural and sensory properties to a wide variety of products. Additionally, they have shown great potential for various engineering applications due to their unique properties, such as their biocompatibility, biodegradability, and tunable mechanical properties [1]. The use of animal proteins such as gelatin and egg-white protein (EWP) has been among the most common practices in the production of these gels; however, there is a growing trend to supplement or even replace animal proteins with plant-based alternatives. The food industry is progressively focusing on sustainability concerns. Recently, plant-based proteins have emerged as a potential solution for the growing nutritional needs of the global population [2]. In addition to their lower production cost, there is a growing trend to reduce livestock production. This trend is driven by the growing interest in plant-based diets and a desire to reduce the environmental impact of food production. However, from a technological perspective, plant proteins have poorer functional properties than animal proteins, such as a low solubility and weak emulsifying and foaming capabilities as well as sub-optimal gelling and extrusion properties [3]. Additionally, their nutritional value is lower, with a poorer amino acid composition and low bioactivity and digestibility. Nevertheless, these limitations can be overcome by developing efficient approaches to produce plant-based gels with improved characteristics, e.g., by the selective mixing of different protein sources. Pumpkin (*Cucurbita pepo* L.) is among the most widely cultivated cucurbit crops in the world. Depending on the region, pumpkin can be produced for decorative purposes, as a culinary ingredient, or as a source of oil from the seeds [4].

After oil extraction, the main byproduct is defatted pumpkin cake. This cake is a rich source of protein, containing up to 65% of this essential nutrient. In addition, it contains carbohydrates, dietary fiber, and many vitamins and minerals [5]. Pumpkin seeds contain four basic groups of proteins; namely, water-soluble albumin, alkali-soluble glutelin, alcohol-soluble prolamin, and salt-soluble globulin. The most abundant are globulins and glutelins [6]. In addition to their good functional and nutritional properties, these proteins have anti-inflammatory, antioxidant, antiviral, and antibacterial activities [7]. Unfortunately, the content of isoleucine, leucine, histidine, and valine in pumpkin-seed protein is too low to make it a good source of essential amino acids. However, pumpkin seeds are a rich source of methionine and lysine [8]. On the other hand, egg-white protein contains all 20 amino acids in proper proportions; therefore, it has an optimal composition and a very high nutritional value. It contains 8.9% leucine, 7.3% valine, and 5.6% isoleucine. Furthermore, egg-white albumin is abundant in branched and aromatic amino acids [9]. It has a huge impact on muscle development as leucine induces a large anabolic response in skeletal muscle [10]. In the light of this, the supplementation of pumpkin proteins with egg-white proteins should produce mixed gels with a rich amino acid composition and an optimal nutritional value.

In food technology, the nutritional value is equally important as the functional properties, which enables a broad spectrum of different commercial products to be produced. Among these, gelation is of special importance. It shapes the product, entraps water and different nutrients, and allows the formation of very different textures. The physicochemical properties of heat-induced gels derived from pumpkin-seed protein isolates have been investigated and compared with those of pea protein and soybean protein isolates [11]. Before the gelation process, the proteins were sonicated or treated at pH 3 or 11. In a native state, pumpkin-seed protein formed the strongest gels among the three proteins. This was probably due to the high hydrophobicity of this protein. Ultrasound or an alkaline treatment induced a larger improvement in the rheological properties of the pumpkin-seed protein than the other proteins. The acid treatment decreased the solubility of all investigated proteins because of an increase in the size of aggregates [11]. Alavi et al. [12] obtained co-aggregates of egg-white protein and hemp-seed protein isolates. The solutions were heated under an alkaline pH and, after cooling, acid-induced gelation was performed by adding glucono-δ-lactone. Increasing the hemp-seed protein content had a negative effect on the rheological properties (storage modulus, loss tangent, creep performance, texture parameters, and fracture stress/strain). Mixed gels with a larger hemp protein content were characterized by decreased microstructural homogeneity. Similar results were observed for dairy/plant-protein mixed gels, which showed a lower gel strength than pure dairy protein gels [13,14]. Plant and dairy proteins form independent networks with smaller interactions between the different protein matrices. Plant proteins have also been shown to compete with dairy proteins for calcium, which influences the gelation process. On the other hand, in the production of real food products, it was found that a partial replacement of horsemeat with an emulsion gel containing pumpkin-seed protein improved the textural properties of semi-smoked sausages [15].

To our knowledge, there is no publication on mixed pumpkin-seed protein/egg-white gels. Thus, the aim of this research was to explore the gelation process of binary mixes of pumpkin-seed and egg-white proteins. The substitution of plant proteins with egg-white proteins would increase the nutritional value of the product, enriching it with essential amino acids. Additionally, the questionable functional properties of pumpkin protein gels could be enhanced by supplementation with egg-white proteins. The combination of plant and animal proteins also offers the potential for cost savings and improved sustainability. Plant proteins are generally less expensive and have a lower environmental impact compared with animal proteins, making them an attractive option for food engineering applications. Last but not least, this is an excellent way to introduce plant-based protein into existing formulations. Consequently, it is essential to understand the structural and mechanical properties of mixed gels containing plant proteins, and how these properties can be tailored by selective mixing.

## 2. Materials and Methods

### 2.1. Materials

Pumpkin-seed protein concentrate was obtained from Medicaline (Ostrówiec, Poland). The protein content was 60.2%. According to the producer, it contained 12.8% fat, 13.0% fiber, 2.5% carbohydrate, and 2.1% minerals. Egg-white albumen of a “low mineral, high gel-type” (88.1% protein) was a gift from the Kewpie Corporation (Tokyo, Japan). The protein concentration of both protein materials was determined by the Kjeldahl method [16].

### 2.2. Sample Preparation

Mixed dispersions of pumpkin-seed protein concentrate/egg-white protein isolate (12/0%, 10/2%, 8/4%, 6/6%, 4/8%, 2/10%, and 0/12%) were obtained in 0.2 M NaCl. The dispersions were put into 10 mm diameter glass tubes and heated in a water bath for 30 min at 80 °C. After heating, the samples were cooled down under tap water and stored at 7 °C for 24 h. Before the measurements, the samples were equilibrated to a temperature of 21 °C. 

### 2.3. Small-Strain Rheology

Gel samples were cut to obtain 2 mm thick discs (10 mm diameter). The viscoelastic properties of the gels were monitored using a Kinexus Lab+ dynamic rheometer (Malvern Instruments Limited, Malvern, UK). The oscillatory measurements were performed at 2 mm gaps in the frequency range of 0.1–10 Hz at 21 °C. All measurements were made at 1% strain, which was in the linear viscoelastic range determined by the strain sweep.

### 2.4. Ultrasonic Viscosity Measurements

The probe was immersed in the gel and the values of viscosity × density (mPas × g/cm^3^) were measured. The measurements were carried out using a ultrasonic viscometer (UNIPAN type 505, Warsaw, Poland). Six measurements were performed to obtain a single average result. 

### 2.5. Compression of the Gels

Gels were cut to obtain 10 mm high cylinders (10 mm diameter). The gels were lubricated with a thin layer of paraffin oil and compressed with a flat probe (φ = 75 mm) [17]. Samples were compressed using a TAXT2i texturometer (Stable Micro Systems, Haselemere, UK) to a 60% deformation at the speed of 0.5 mm/s. The hardness of the gels was presented as the maximum force of compression in grams force. Eight samples were compressed for each factor combination and the results were presented as the arithmetic mean.

### 2.6. Water Activity

Water activity (aw) was measured using an AWMD-10 water activity meter (NAGY, Gäufelden, Germany) with an accuracy of ±0.001 aw per unit. Before each measurement, the device was calibrated according to a special humidity standard (95% HR). Measurements were made at 20 °C.

### 2.7. Gel Surface Topography

The gel surface (0.9 × 1.3 mm) was investigated using a GT Contour Surface Metrology optical profilometer (Veeco, Tucson, AZ, USA). The surface topography was characterized in the range from sub-nanometer to 1 mm. The quadratic mean of the surface roughness, i.e., Rq, was calculated using the Vision64 (Veeco, Tucson, AZ, USA) computer program.

### 2.8. Polarizing Optical Microscopy

The gel surface microstructure was observed using a Eclipse E600 Pol polarizing optical microscope (Nikon, Tokyo, Japan). The glass slips were cleaned using isopropyl alcohol and a cotton pad. Finally, they were blow-dried with nitrogen. The preparation was observed on a computer monitor coupled to a microscope and equipped with NIS-Elements AR 4.50 software (Nikon, Tokyo, Japan).

### 2.9. Scanning Electron Microscopy (SEM)

Gels were fixed by immersion in a 2.5% glutaraldehyde solution in a 0.1 M sodium cacodylate buffer. The samples were dehydrated in serial dilutions of ethanol and acetone and dried at the critical point in liquid carbon dioxide. The preparations were coated with gold using a EMITECH K550X vacuum evaporator (Emitech, Ashford, UK). The preparations were viewed and photographed using a VEGA II LMU scanning electron microscope (Tescan, Canberra, ACT, Australia; Warrendale, PA, USA).

### 2.10. Fourier Transform Infrared Spectroscopy

Infrared absorption spectra were recorded with a Vertex 70 FTIR spectrometer (Bruker, Billerica, MA, USA). The attenuated total reflection (ATR) configuration was used with 10 internal reflections of the ZnSe crystal plate (45° cut). Typically, 16 scans were taken at a resolution of 2 cm^−1^. The spectra were obtained in the region between 4000 and 600 cm^−1^. The instrument was purged with N_2_ for 40 min before and during the measurements. The ZnSe crystal plate was cleaned with ultra-pure organic solvents. Curve fittings were analyzed by OriginPro 9.0 software. All measured bands were shifted to the same level of background before analysis.

### 2.11. Statistical Analysis

The statistical analysis (standard deviation and analysis of variance) of the results was performed using the statistical program STATISTICA 5.0 PL (StatSoft Polska, Warsaw, Poland). The significance of differences between the means was determined using Tukey’s test at a confidence level of *p* ≤ 0.05, based on the least significant difference.

## 3. Results and Discussion

### 3.1. Rheological Properties

**Figure 1:**
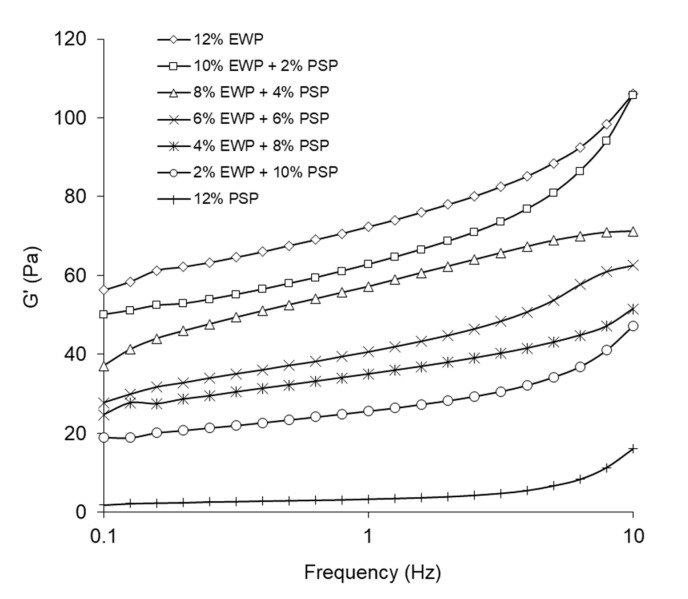
Influence of frequency on storage modulus of mixed gels.

**Figure 2:**
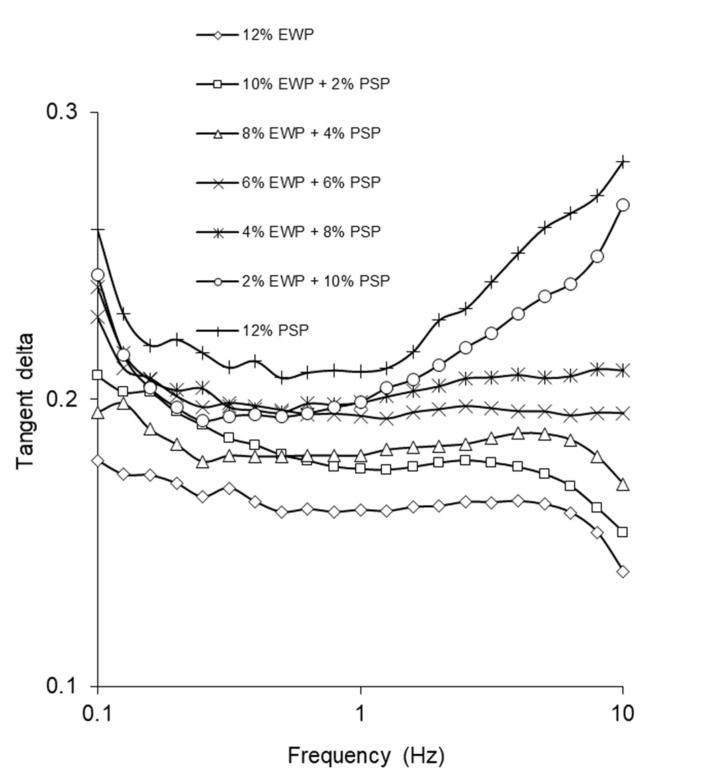
Influence of frequency on tangent delta of mixed gels.

Figure 1 presents the frequency sweep versus the storage modulus, i.e., G′, of the investigated gels. For all studied gels, the storage modulus increased with the frequency. The substitution of pumpkin-seed protein with egg-white protein caused an increase in G′; thus, the gels containing pumpkin-seed protein were less elastic. Rafe et al. [14] observed increased values of the storage modulus after increasing the proportion of whey protein in mixed gels with rice-bran protein. For gels with a higher concentration of egg-white protein than pumpkin-seed protein, lower values of tangent delta were obtained (Figure 2).

This indicated that for gels with a higher content of egg-white protein, the elastic properties governed over the viscous ones. Furthermore, an inverse relationship was observed between tangent delta and frequency in gels with a higher EWP content. An increase in frequency caused a decrease in tangent delta. According to Debora’s law, when gels are subjected to a shorter time of deformation at a higher frequency, they tend to exhibit more elastic behavior [18]. An opposite situation was observed for the gels with a lower concentration of EWP (0–4%). At higher frequencies, an increase in the tangent delta values was noted (Figure 2). The gel structure with a higher concentration of pumpkin-seed protein weakened at a higher frequency (higher energy input). An increase in frequency caused an increase in the storage modulus, but some bonds were broken and the viscous component became more evident. The values of tangent delta remained quite low (below 0.3), indicating the elastic nature of the gels.

The bulk properties of the mixed gels were also investigated using an ultrasonic viscometer. This device works by transmitting ultrasonic waves through a material. When an ultrasonic wave propagates through a solid, the material tends to slow it down, causing the damping of the wave. The phenomenon of wave attenuation is the result of several mechanisms of absorption, mainly due to viscosity [19]. A waveguide is used to remotely transmit the ultrasonic waves from a shear piezoelectric transducer into the material. At the waveguide-measured material interface, a guided wave shear mode is applied to measure the material viscosity. The energy of the reflected ultrasonic wave depends on the frequency, viscosity, and density of the material and the waveguide properties (density and shear modulus). From the relationship between the reflection coefficient of a wave at a waveguide-measured material interface and the acoustic impedance of the material, the product of the density and viscosity can be obtained. By measuring the density of the material, the viscosity value can be calculated [20]. 

**Figure 3:**
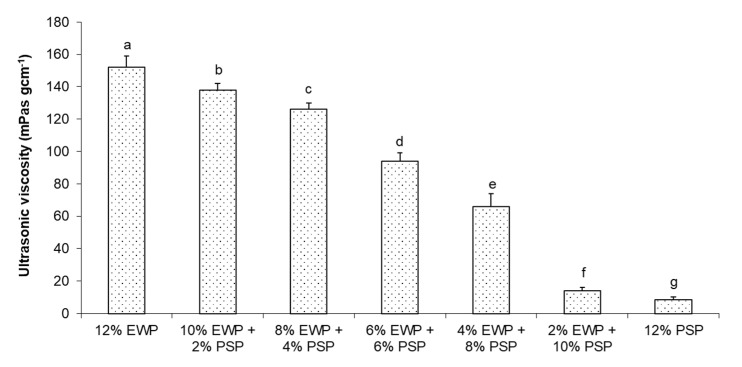
Ultrasound viscosity of mixed gels. Means with different letters (**a**–**g**) were significantly different (*p* ≤ 0.05).

The viscosity values obtained for the mixed gels are depicted in Figure 3. A decrease in ultrasound viscosity was observed as the concentration of egg-white protein decreased.

**Figure 4:**
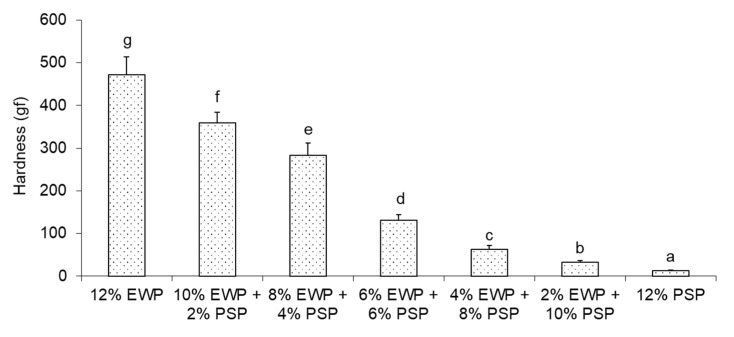
Hardness of mixed gels. Means with different letters (**a**–**g**) were significantly different (*p* ≤ 0.05).

The same relationship was observed for the hardness of the gels (Figure 4). Interestingly, an exponential correlation was found between ultrasound viscosity and hardness (R^2^ = 0.97).

**Figure 5:**
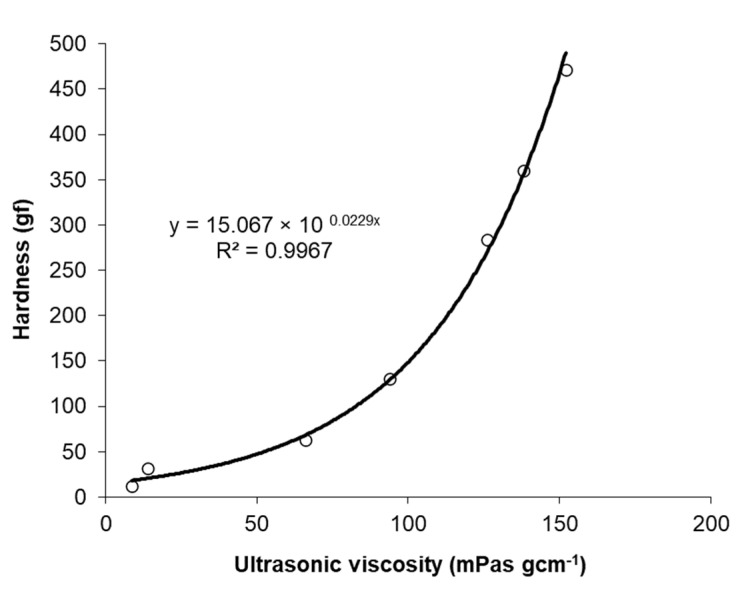
Correlation between ultrasound viscosity and hardness for PSP/EWP mixed gels.

Hardness is a measure of the resistance of a material to an applied force. Force is applied by means of compression tension exerted on the surface of a sample. Acoustic impedance is a measure of the resistance of the material to sound-wave propagation. In our measurements, the waveguide was in the form of a thin metal probe (3 × 1 mm) and the hardness was measured by the compression of a 10 mm diameter sample. Comparing the response of the gels to sound-wave propagation and compression, a higher increase in resistance was observed in the latter. This may explain the exponential correlation observed between ultrasonic viscosity and hardness. (Figure 5). 

**Figure 6:**
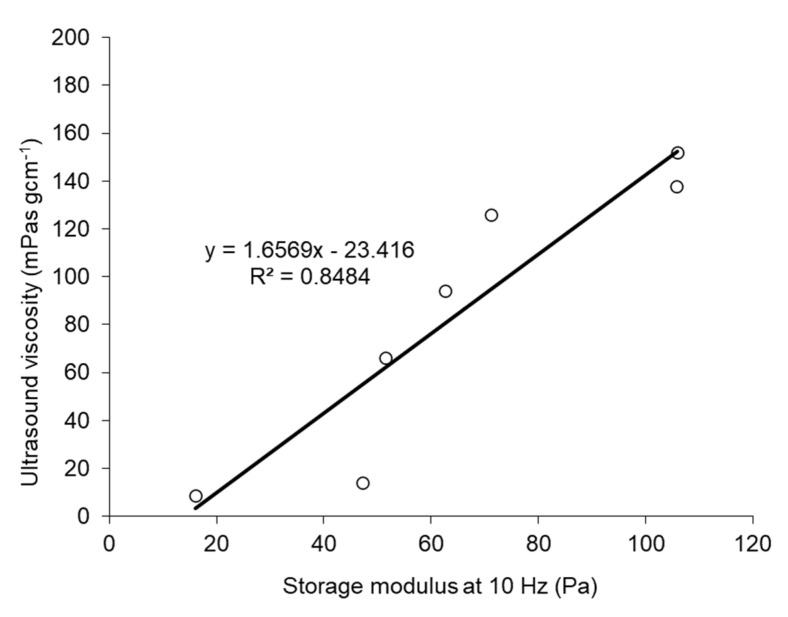
Correlation between storage modulus at 10 Hz and ultrasound viscosity for PSP/EWP mixed gels.

Additionally, a linear correlation was noted between ultrasound viscosity and the storage modulus at 10 Hz (R^2^ = 0.90) (Figure 6). The linear correlation may be attributed to the fact that both methods were based on small-strain deformations. This relationship suggested that the measured viscoelastic properties were consistent across these methods, indicating that the observed properties were intrinsic to the material being studied. Based on the mechanical response data obtained, it was concluded that the strength and stiffness of the mixed gels increased with a decreasing plant protein fraction. Similar results have been reported for whey- and soy-protein mixed gels [21].

### 3.2. Microstructure

**Figure 7:**
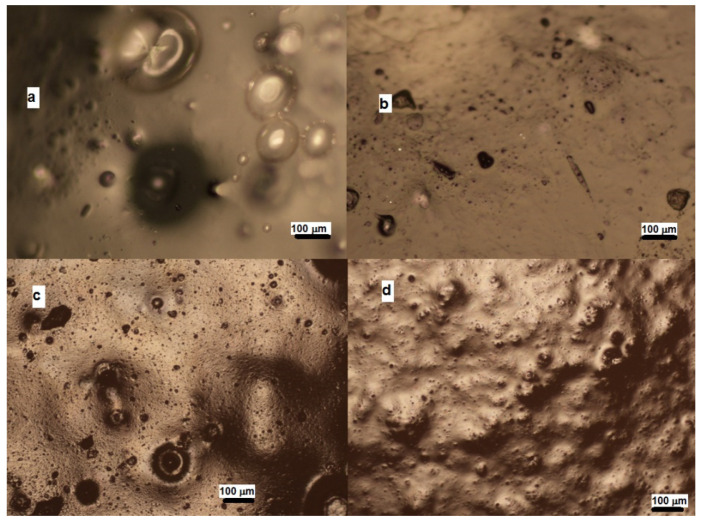
Polarizing optical microscopy view of mixed gels: (**a**) 12% EWP; (**b**) 8% EWP and 4% PSP; (**c**) 4% EWP and 8% PSP; (**d**) 12% PSP.

Figure 7 shows the microstructure of the investigated gels obtained by polarizing optical microscopy. Apparently, gels with a larger concentration of egg-white protein had a more homogenous structure with a smoother surface (Figure 7a,b). Increasing the pumpkin-seed protein content resulted in a rougher surface with a more particulate microstructure (Figure 7c,d).

**Figure 8:**
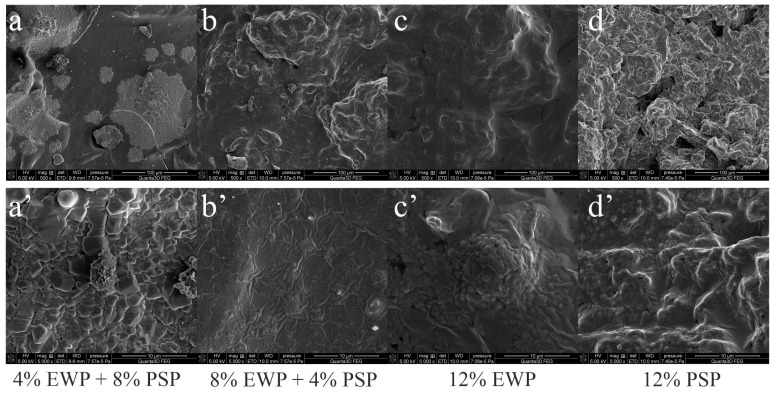
Scanning electron microscopy of mixed gels. (**a**,**a’**)—4% egg-white protein (EWP) mixed with pumpkin seeds protein (PSP), (**b**,**b’**)—8% EWP + 4% PSP, (**c**,**c’**)—12% EWP, (**d**,**d’**)—12% PSP. Images were obtained at different magnifications (top row 500×, bottom row 5000× respectively).

This was confirmed by the scanning electron microscopy images, which revealed further details at a higher magnification (Figure 8). The substitution of egg-white protein with pumpkin-seed protein formed a microstructure in which the globular structures of the plant protein formed “islands” within the egg-white protein gel matrix, resembling a model of “islands in the sea” (Figure 8b,c). A higher concentration of pumpkin-seed protein resulted in the formation of more “islands” in a smooth “sea” of egg-white protein gel. Figure 8c’,d’ show the microstructure of the pure egg-white and pumpkin-seed protein gels, respectively. They were observed at a very high magnification. The egg-white protein gel microstructure was fine-stranded (Figure 8c’) and the pumpkin-seed protein gel microstructure was particulate. This could be related to the insoluble fraction of pumpkin-seed protein. The solubility of pumpkin-seed proteins depends on the pH and drastically increases at a pH above 6, reaching maximal values close to 80% at around pH 7 [22]. This suggested that at least 20% of the pumpkin-seed protein was insoluble in the obtained mixed gels, which may have resulted in the observed particulate microstructure.

**Figure 9:**
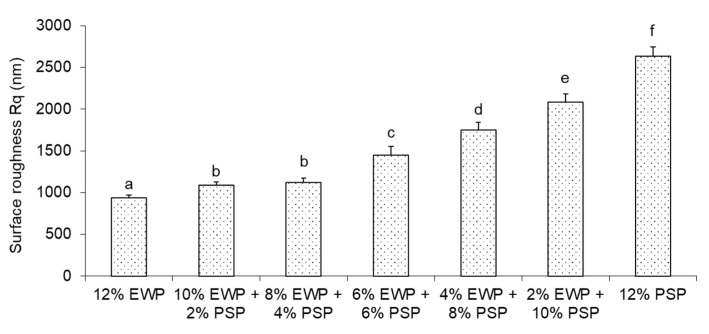
Surface roughness of mixed gels. Means with different letters (**a**–**f**) were significantly different (*p* ≤ 0.05).

The increased roughness of the gels with a higher concentration of pumpkin-seed protein was corroborated by the roughness parameter obtained from the optical profilometer (Figure 9). This parameter showed an upward trend; it increased from 1080 nm for the mixed gel with the lower concentration of plant protein to 2200 nm for the one with the higher concentration. This type of microstructure with plant-protein aggregates wrapped in egg-white protein gel was previously observed in soybean protein/egg-white mixed gels [23]. The phenomena of non-homogeneity observed in mixed gels at the microscale could potentially be elucidated by looking at their microstructure at a nanoscale. Egg-white gel has a very homogenous microstructure with very small particles (Figure 8c’). This phenomenon can be attributed to the fact that the egg-white protein isolate used in the research was an experimental preparation obtained by Kewpie Egg Corporation, which contained a very small concentration of ions [24,25]. Heat-induced gelation in the 0.2 NaCl solution resulted in a fine-stranded gel being obtained (Figure 8c’) [26]. In the same conditions, the pumpkin-seed protein formed a gel with a particulate microstructure (Figure 8d’). Kuang [27] investigated mixed gels of egg-white proteins and pea proteins. The gels comprised a fine-stranded network of egg-white proteins with aggregates of pea proteins. The structure of the egg-white protein network was formed by strong disulfide bonds and hydrophobic interactions; hydrogen bonds were mostly responsible for the weak microstructure of the pea-protein aggregates. In mixed gels, the egg-white protein matrix was accompanied by the formation of protein aggregates, which may have been pure pea protein aggregates or mixed with some egg-white proteins [27].

### 3.3. Fourier Transform Infrared Spectroscopy

**Figure 10:**
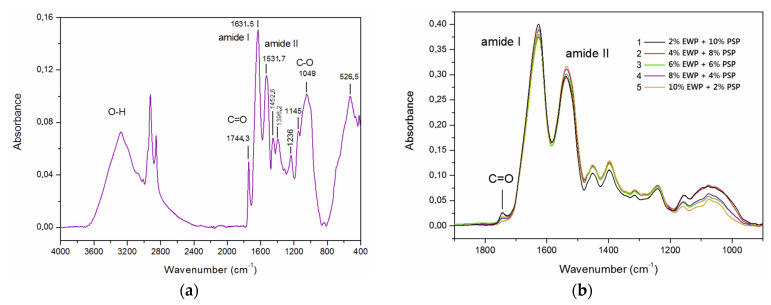
FRIR absorption spectra of investigated gels: (**a**) spectrum noted for 12% PSP gel; (**b**) spectra for mixed gels.

Figure 10 presents the infrared absorption spectra for the investigated gels. Major absorption bands were noted at 1026 cm^−1^ (primary amine; C-N stretch), 1655 cm^−1^ (amide I; C=O stretch), 2927 cm^−1^ (methyl, methylene, and methine; C-H stretch), and 3362 cm^−1^ (O-H and N-H stretching vibrations). Spectral peaks between 1655 and 1657 cm^−1^ corresponded with the characteristic band of amine I (Figure 10b). The vibration of amide I represents the secondary structure of the protein skeleton, which is often used for the quantitative analysis of different secondary structures. The bands between 3349 and 3374 cm^−1^ represent the overlapping of the stretching vibrations of the O-H and N-H groups [28].

In the 12% PSP spectrum (Figure 10a), two characteristic bands assigned to amide I at 1631 cm^−1^ and amide II at 1532 cm^−1^ were shifted toward smaller wavelengths compared with the literature values. It indicated that there were interactions between pumpkin-seed protein and other ingredients present in the pumpkin-seed protein concentrate [29,30]. This was probably connected with the formation of hydrogen bonds and even new functional groups. New hydrogen bonds between the side chains of amino acids determine the shape of a protein, showing the secondary structure of the protein. Hydrogen bonding can involve both amide I bonds; i.e., C=O and N-H [31]. The band at 1743 cm^−1^ corresponded with the stretching vibrations of the C=O group. The band at 1396 cm^−1^ was assigned to the symmetric CH_3_ bending of the methyl groups of proteins. Bands observed at 2920 and 2849 cm^−1^ could be attributed to the asymmetry and symmetry stretching bonds of -C-H in the CH_2_ groups, respectively. The bands noted at 1453 cm^−1^ were attributed to the bending (scissoring) of the -C-H bonds in the CH_2_ and CH_3_ groups, whereas the bands observed at 1236 cm^−1^ corresponded with the stretching and bending of the -C-O bonds in the -CH_2_- groups. Bands observed at 1049 cm^−1^ and 1145 cm^−1^ were assigned to the stretching vibrations of hydrogen bonds in the C-OH groups [32]. The intensity of these bands decreased with an increase in the content of egg-white protein (Figure 10b). This could be explained by the fact that pumpkin-seed protein concentrate contains other constituents that interact through hydrogen bonds with pumpkin-seed protein, and these constituents were probably replaced by pure egg-white protein. Additionally, the amide II intensity also changed with the substitution of pumpkin-seed protein with egg-white protein (Figure 10b). This band showed the coupling between the water and protein residuals in the heating process to obtain the protein gels, and could be associated with changes in the secondary structure of proteins. A decrease in the intensity of this band with an increase in the concentration of pumpkin-seed protein suggested that the protein secondary structure was evolving more toward a linear chain of amino acids in comparison to the egg-white protein [28].

### 3.4. Water Activity

Water activity (aw) is the ratio of vapor pressure and vapor saturation pressure at the same temperature. This measurement is used to control the quality and stability of food in terms of microorganism growth, enzymatic reaction rates, and physical properties.

**Figure 11:**
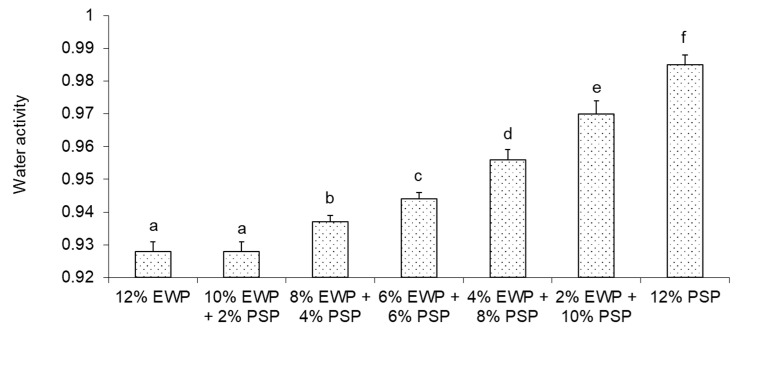
Water activity of mixed gels. Means with different letters (**a**–**f**) were significantly different (*p* ≤ 0.05).

Increasing the concentration of egg-white protein caused a decrease in water activity (Figure 11). Similar results were found by Kusio et al. [33] for high-protein fat-free dairy desserts. The water activity significantly decreased with a higher concentration of whey protein concentrate. The supplementation of pumpkin-seed protein with egg-white protein caused a decrease in water activity from 0.985 to 0.928. This could have had an influence on the microbiological stability of the obtained gels. For instance, Theys et al. [34] found that lowering the aw from 0.990 to 0.970 caused a decrease in the growth rate of *Salmonella Typhimurium* in gelatin gels.

Very interesting correlations were found between the water activity and rheological properties of the obtained gels. Stronger gels had a lower water activity. A linear correlation was found between the water activity and storage modulus at 10 Hz (R^2^ = 0.91), and between the water activity and ultrasound viscosity (R^2^ = 0.96); an exponential correlation was found between the water activity and hardness (R^2^ = 0.99) (Supplementary Materials). Agoda-Tandjawa et al. [35] found that water activity measurements could be used as a tool to evaluate the rheological properties of sewage sludge and to predict its mechanical and structural properties during dewatering and aging. They noted a decrease in water activity with an increase in the storage modulus and loss modulus. Water activity is a measure of how water molecules are bound to the supramolecular structure of a material. The supplementation of pumpkin-seed protein with egg-white protein resulted in gels with a stronger microstructure and better binding with water. Gels obtained from more soluble egg-white protein formed a more homogenous matrix capable of holding more water. Materials characterized by a higher water-holding capacity have a smaller water activity [36]. Pumpkin-seed protein was less soluble and formed a particulate gel structure with larger pores (Figure 8c’,d’), and had a smaller capacity to hold water.

## 4. Conclusions

The substitution of plant protein with egg-white protein is necessary to increase the nutritional value of the product, enriching it with essential amino acids. The addition of egg-white protein enhanced the rheological properties of the mixed gels. Gels were more elastic and more resistant to breaking structure. An increased concentration of egg-white protein caused a decrease in water activity to a level that could have had an impact on the microbiological stability of the obtained gels. The supplementation of pumpkin-seed protein with egg-white protein resulted in more homogenous gels with a stronger microstructure and better binding with water. Mixed plant-protein/animal-protein gels are an attractive proposition for the food industry to obtain new products with a novel texture, good nutritional value, and microbiological stability.

## Data Availability

The data are contained within the article.

## Supplementary Materials

The following supporting information can be downloaded at: https://www.mdpi.com/article/10.3390/foods12102030/s1, Figure S1: Correlation between water activity and storage modulus at 10 Hz; Figure S2: Correlation between water activity and ultrasound viscosity; Figure S3: Correlation between water activity and hardness.
