# Supplementary material for: Co-Gelation of Pumpkin-Seed Protein with Egg-White Protein
Source: Foods. 2023 May 17;12(10):2030. doi: 10.3390/foods12102030 (PMC10217760; doi:10.3390/foods12102030)
Supplement: Supplementary file 1 [file foods-12-02030-s001.zip › foods-2393539-supplementary.pdf]

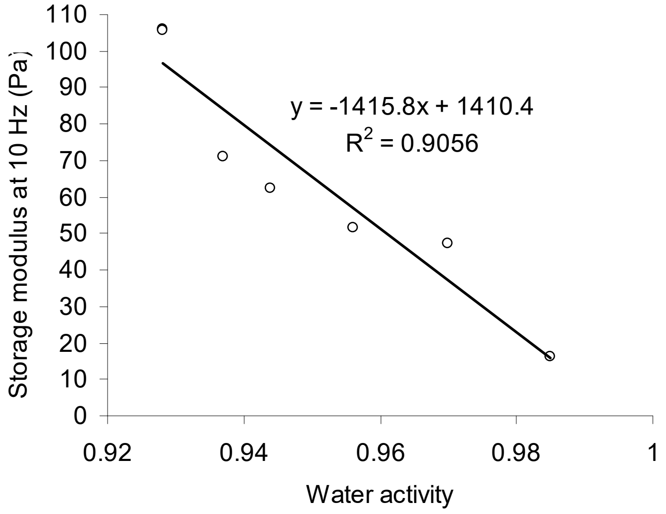

Figure S1. Correlation between water activity and storage modulus at 10 Hz.

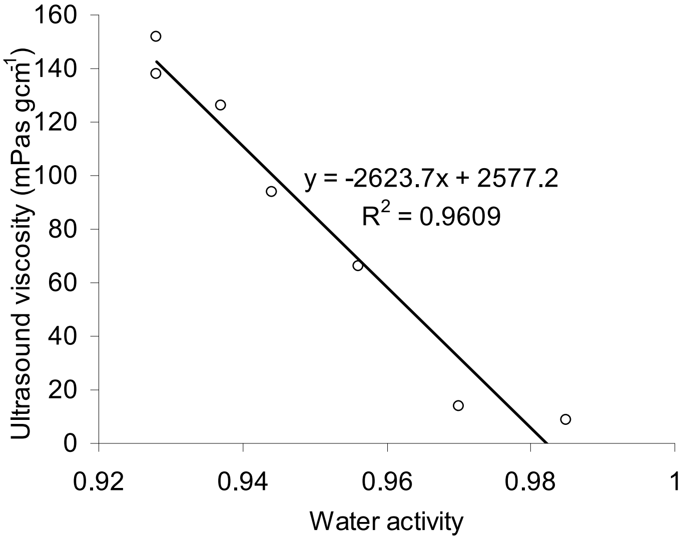

Figure S2. Correlation between water activity and ultrasound viscosity.

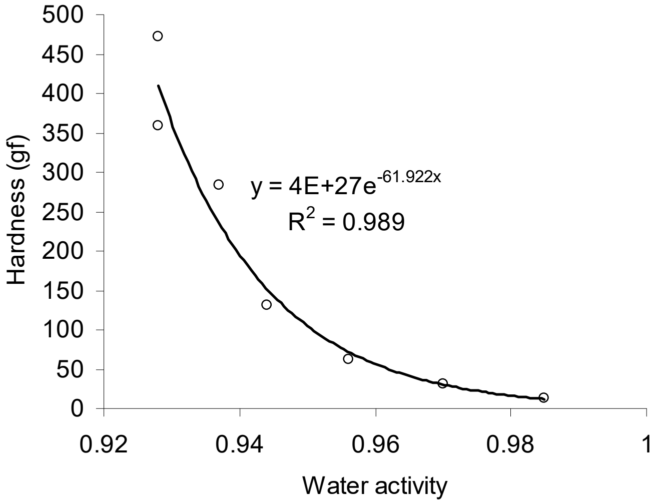

Figure S3. Correlation between water activity and hardness.
